# Aluminum scandium nitride thin-film bulk acoustic resonators for 5G wideband applications

**DOI:** 10.1038/s41378-022-00457-0

**Published:** 2022-11-29

**Authors:** Yang Zou, Chao Gao, Jie Zhou, Yan Liu, Qinwen Xu, Yuanhang Qu, WenJuan Liu, Jeffrey Bo Woon Soon, Yao Cai, Chengliang Sun

**Affiliations:** 1 The Institute of Technological Sciences, Wuhan University, 430072 Wuhan, China; 2 Hubei Yangtze Memory Laboratories, 430205 Wuhan, China; 3 School of Microelectronics, Wuhan University, 430072 Wuhan, China

**Keywords:** Electrical and electronic engineering, Structural properties

## Abstract

Bulk acoustic wave (BAW) filters have been extensively used in consumer products for mobile communication systems due to their high performance and standard complementary metal-oxide-semiconductor (CMOS) compatible integration process. However, it is challenging for a traditional aluminum nitride (AlN)-based BAW filter to meet several allocated 5G bands with more than a 5% fractional bandwidth via an acoustic-only approach. In this work, we propose an Al_0.8_Sc_0.2_N-based film bulk acoustic wave resonator (FBAR) for the design of radio frequency (RF) filters. By taking advantage of a high-quality Al_0.8_Sc_0.2_N thin film, the fabricated resonators demonstrate a large *K*_*eff*_^*2*^ of 14.5% and an excellent figure of merit (FOM) up to 62. The temperature coefficient of frequency (TCF) of the proposed resonator is measured to be −19.2 ppm/°C, indicating excellent temperature stability. The fabricated filter has a center frequency of 4.24 GHz, a −3 dB bandwidth of 215 MHz, a small insertion loss (IL) of 1.881 dB, and a rejection >32 dB. This work paves the way for the realization of wideband acoustic filters operating in the 5G band.

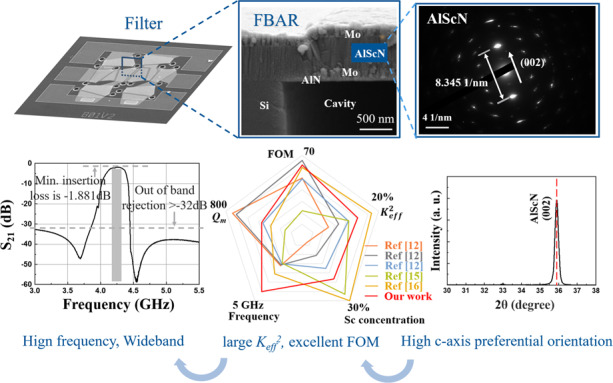

## Introduction

Recently, mobile communication systems have continued to demand a high data rate and great mobility^1–3^. These trends are increasing the need for filters with higher frequencies and wider bandwidths^4^. Emerging 5G, Wi-Fi and 4G LTE communication standards are driving up frequencies from the traditional bands (below 2.6 GHz) to as high as 5 GHz to accommodate wider system bandwidths. Microelectromechanical system (MEMS) filters, such as surface acoustic wave (SAW) and bulk acoustic wave (BAW) filters, are promising candidates for operation at new radio frequency (RF) band sections. However, the SAW filter technology used for current mobile communication systems can hardly achieve frequencies above 3 GHz due to the degraded filter performance and several fabrication problems caused by the very thin and delicate IDT electrode patterns^5^. BAW filters can provide low insertion loss (IL), good selectivity, and high power handling and are preferred in higher frequency applications^6–8^.

Two types of technologies, including film bulk acoustic wave resonators (FBARs) and solidly mounted resonators (SMRs), have been used to manufacture thin film BAW filters^1,8^. SMR devices require a Bragg reflector, preferably patterned with thin-film layers with alternating low and high acoustic impedances. This technology is deemed superior to FBARs in terms of mechanical robustness and power handling. However, the disadvantage of SMRs is an increase in the number of fabrication process steps that should be controlled in terms of film thickness and film properties. For the FBAR, an air cavity is created between the bottom electrode and the carrier wafer. The air/electrode interface can trap more acoustic waves between the electrodes than the Bragg quarter-wavelength acoustic mirror, thus obtaining better effective electromechanical coupling (*K*_*eff*_^2^). Furthermore, the FBAR provides a slightly higher Q due to the lack of additional reflector layers in which the acoustic wave can be attenuated or can escape.

Aluminum nitride (AlN) is utilized in various generations of telecommunications technology due to its high longitudinal sound velocity *v* (11,354 m/s), low temperature coefficient of frequency (TCF, −25 ppm/°C), and low acoustic and dielectric losses^9–11^. Using an optimized FBAR design and a careful choice of materials and nonpiezoelectric layer thickness, the *K*_*eff*_^2^ of the FBAR can be boosted to values of ~7%^12^. However, new materials with higher electromechanical coupling render potential choices, resulting in relaxed device designs and fabrication margins. Al_1-x_Sc_x_N (AlScN) is one of those materials, which can be deposited through the same means as AlN, that shows an increase in the piezoelectric coefficient. For example, the piezoelectric coefficient *d*_33_ of Al_0.57_Sc_0.43_N is up to five times larger than that of pure AlN^13–15^. Moreira et al.^12^ presented an Al_0.85_Sc_0.15_N-based FBAR with a resonant frequency of 2.15 GHz and a *K*_*eff*_^2^ of 12.07%. Wang et al.^16^ demonstrated a switchable Al_0.7_Sc_0.3_N-based FBAR utilizing the newly discovered ferroelectric behavior of Al_0.7_Sc_0.3_N thin films.

In this work, we report an Al_0.8_Sc_0.2_N-based FBAR for the design of acoustic filters. By taking advantage of the high-quality Al_0.8_Sc_0.2_N film, the FBAR presents a large *K*_*eff*_^2^ of 14.5% and an excellent figure of merit (FOM) of 62. Furthermore, the Mason model is used to extract the important intrinsic material parameters of the Al_0.8_Sc_0.2_N film. Subsequently, high- and low-temperature probe stations are employed to study the temperature characteristics of the Al_0.8_Sc_0.2_N-based FBAR. Finally, filters using the Al_0.8_Sc_0.2_N film exhibit a center frequency of 4.24 GHz, a −3 dB bandwidth of 215 MHz, a small IL of 1.89 dB, and a rejection above 32 dB. The proposed Al_0.8_Sc_0.2_N-based FBAR filters show potential for 5G wideband applications.

## Design and fabrication

**Fig. 1:**
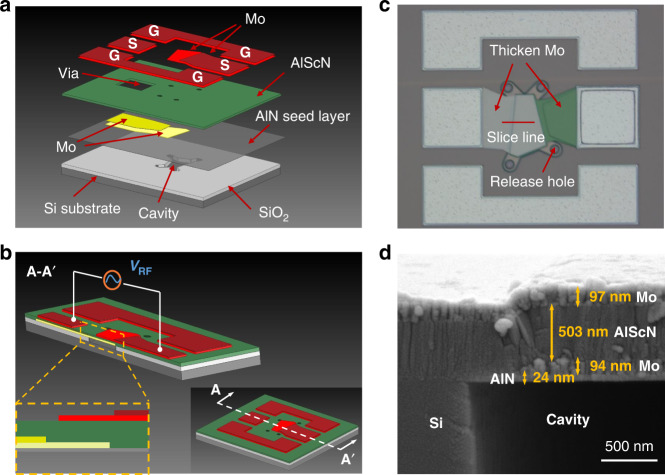
Al0.8Sc0.2N-based FBAR. **a** Explosive view of the layered structure of the designed resonator. **b** The practically enlarged section view of the resonator. **c** Optical microscope of the fabricated resonator. **d** SEM image of the cross-sectional view of the fabricated resonator.

**Fig. 2:**
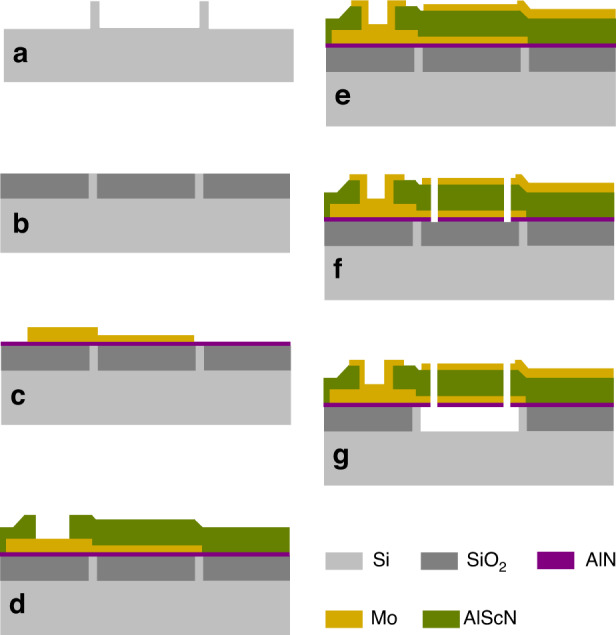
Main process steps for the fabrication of AlScN-based FBARs. **a** Forming isolation walls by etching the Si substrate. **b** SiO_2_ deposition and chemical mechanical polishing (CMP). **c** Bottom Mo dual deposition and pattern. **d** Al_0.8_Sc_0.2_N film deposition and etched by inductively coupled plasma (ICP). **e** Top Mo dual deposition and pattern. **f** Opening the release window. **g** Releasing the cavity by VHF.

The designed piezoelectric film bulk acoustic resonator, illustrated in Fig. 1, is composed of six thin films grown on a Si substrate (725 *μ*m thick). Two additional Mo layers of 120 nm and 37 nm outside the cavity are designed on the top and bottom electrodes, respectively, to reduce electrode resistance. As shown in Fig. 1(a) and (b), the top and bottom electrode layers are patterned to quadrilaterals, and electrical connection strips are used to conduct electrical signals to the pads (S). When an RF signal is applied between the two electrodes of the resonator, longitudinal bulk acoustic waves are excited in the piezoelectric film. Figure 1(d) shows the scanning electron microscopy (SEM) view. Its cross-sectional stack at the slice line is exhibited in Fig. 1(c). On the basis of this design, the proposed AlScN-based FBAR is fabricated utilizing a complementary metal-oxide-semiconductor (CMOS)-compatible microfabrication process involving a combination of seven photolithography masks^17–19^. The detailed fabrication process is demonstrated in Fig. 2. The fabrication process starts with etching Si to form the isolation walls, which are used to define the cavity. Chemical mechanical polishing (CMP) is used to provide a flat surface for the deposition of the bottom Mo electrode and piezoelectric AlScN film^20^. In particular, an isolation wall instead of a swimming pool is used to reduce the difficulty of CMP due to the smaller polished area. Next, a 25 nm-thick AlN seed layer and 100 nm-thick bottom electrode Mo are deposited. Moreover, an additional 120 nm-thick Mo layer is formed on the top surface of the bottom electrode at the position where the opening for the bottom electrode electrical contact will be formed. The presence of the additional Mo layer ensures that the bottom electrode will always retain sufficient thickness after etching the openings in the piezoelectric layer. Then, a 500 nm-thick piezoelectric Al_0.8_Sc_0.2_N film is deposited and etched by inductively coupled plasma (ICP) etching to open the bottom electrode. A 100 nm-thick Mo layer is deposited and patterned as the top electrode. Subsequently, a mass loading layer of 37 nm-thick Mo film is deposited and etched above the structure. Finally, the release window is opened, and the whole device is released by a VHF isotropic etcher.

## Results and discussion

**Fig. 3:**
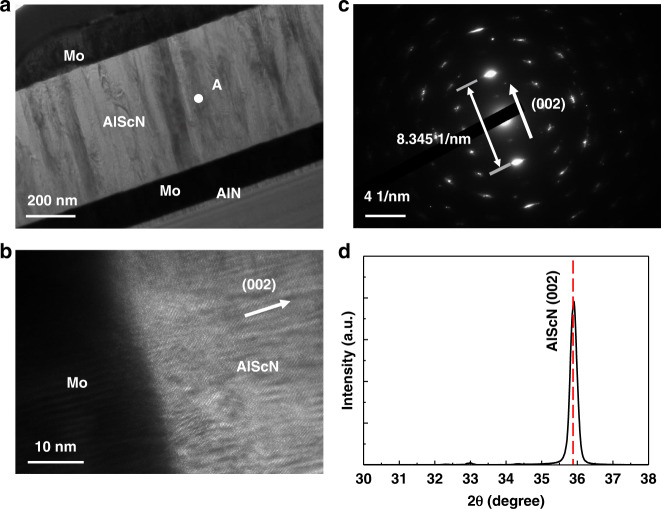
Al_0.8_Sc_0.2_N film characterization. **a** Cross-sectional TEM image of the fabricated FBAR. **b** High-resolution TEM image of the Al_0.8_Sc_0.2_N and Mo interface. **c** Electron diffraction pattern of Al_0.8_Sc_0.2_N in region A. **d** XRD pattern of the Al_0.8_Sc_0.2_N film on a linear scale.

The cross-sectional transmission electron microscopy (TEM) images are shown in Fig. 3(a) and (b). The Al_0.8_Sc_0.2_N film exhibits a good (002) c-axis preferential crystal orientation, which is preferred for achieving a high Q value for the FBAR^21,22^. Figure 3(c) shows the electron diffraction pattern of the Al_0.8_Sc_0.2_N film in region A of Fig. 3(a). The distance between the two diffracted spots closest to the central spot is 8.345 1/nm, corresponding to a lattice plane (002) spacing of 0.240 nm, which is slightly smaller than that of AlN (0.249 nm). In addition, X-ray diffraction (XRD) 2θ/ω scans of the Al_0.8_Sc_0.2_N films include only diffraction maxima attributed to Al_0.8_Sc_0.2_N (002), confirming that the Al_0.8_Sc_0.2_N layer has a single out-of-plane crystallographic orientation.

**Fig. 4:**
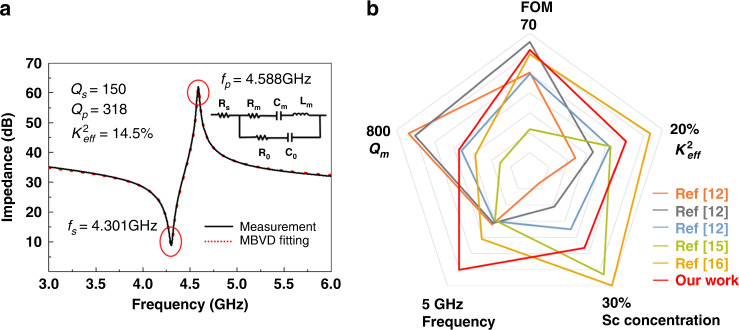
Experimental results of Al_0.8_Sc_0.2_N-based FBAR. **a** Measured and MBVD fitted plot for Al_0.8_Sc_0.2_N-based FBAR. **b** Comparison with different studies regarding the performance of AlScN FBAR^12^^,15,16^.

The frequency response of Al_0.8_Sc_0.2_N-based FBARs is tested in air using a Cascade Microtech GSG probe station (Cascade, America) in conjunction with a network analyzer (N5222B, Agilent Technology). The measured impedance response of the resonator is shown in Fig. 4(a). The *K*_*eff*_^2^ of 14.5%, *Q*_*s*_ of 150 and *Q*_*p*_ of 318 are evaluated by^23^

1 $$K_{eff}^2 = \frac{{\pi ^2}}{4}\frac{{f_s}}{{f_p}}\frac{{f_p - f_s}}{{f_p}}$$

2 $$Q_{s,p} = \frac{{f_{s,p}}}{{\Delta f_{s,p} - 3dB}}$$

where *f*_*s*_ and *f*_*p*_ are the series and parallel resonant frequencies, respectively. The high *K*_*eff*_^2^ value of the FBAR is directly related to the enhanced piezoelectric performance with increased Sc content^24^. The measured result is modeled with the modified Butterworth-Van Dyke (MBVD) model with the circuit shown in the insets in Fig. 4(a). The model consists of a static capacitor *C*_*0*_, electrode resistance *R*_*s*_, dielectric loss *R*_*0*_ and the motional branches, including a motional resistor *R*_*m*_, a motional inductor *L*_*m*_, and a motional capacitor *C*_*m*_^25^. The three motional elements in the MBVD circuit can be defined as

3 $$C_m = C_0\left( {\left( {\frac{{f_P}}{{f_S}}} \right)^2 - 1} \right)$$

4 $$L_m = \frac{1}{{{{{\mathrm{\omega }}}}_s^2C_m}}$$

5 $$R_m = \frac{1}{{{{{\mathrm{\omega }}}}_sC_mQ_s}}$$

where *ω*_*s*_ is the angular resonant frequency (*ω*_*s*_ = 2π*f*_*s*_). The MBVD model fits the measured data with high accuracy, and using Eq. 6, *Q*_*m*_ is calculated to be 424.7. The fitted components are shown in Table 1. Remarkably, due to the thin electrode, the electrode resistance *R*_s_ is 1.92 Ω, which is larger than the other two losses. *Q*_*s*_ is strongly influenced by ohmic loss *R*_s,_ while at *f*_*p*_, the loss is negligible, resulting in *Q*_*s*_ being almost half the value of *Q*_*p*_. Figure 4(b) shows the comparison of the performances of the fabricated FBAR in this work and previous studies^12,15,16^. The resonator presents the advantage of having a high frequency of 4.3 GHz and an excellent FOM of 62, which is essential for fabricating a 5G wideband filter.

6 $$Q_m = \frac{1}{{R_m}}\sqrt {\frac{{L_m}}{{C_m}}}$$

**Table 1:** MBVD model parameters.

MBVD Model	*R*_*s*_ (Ω)	*R*_*m*_ (Ω)	*C*_*m*_ (pF)	*L*_*m*_ (nH)	*C*_*0*_ (pF)	*R*_*0*_ (Ω)	*Q*_*m*_
Value	1.92	0.84	0.10	13.20	0.75	0.85	424.7

**Fig. 5:**
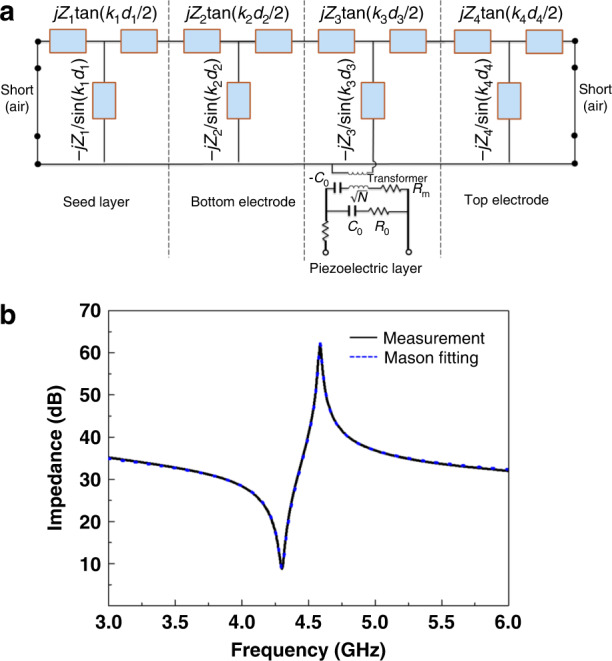
Comparison between simulated value of Mason model and measured result. **a** Equivalent Mason circuit model of an FBAR structure. **b** The fitted impedance curve of the Mason model and the measured impedance curve.

To further assess the Al_0.8_Sc_0.2_N properties, the Mason model is used to fit the measured data to extract important intrinsic material parameters. Figure 5(a) shows the Mason model of the FBAR derived from the one-dimensional acoustic wave equation^23^. In this model, *Z* is the characteristic acoustic impedance, *k* (*k* *=* 2*πf/v*) is the wavenumber, *d* is the thickness of each layer, and $N = k_3d_3/(2\pi fk_t^2C_0Z_3)$, where $k_t^2$ is the intrinsic electromechanical coupling factor of the Al_0.8_Sc_0.2_N film. The bulk material constants for the Mo electrodes and the AlN seed layer are used in this extraction procedure. The layer thicknesses are measured in advance by SEM, as reported in Section II. The acoustic impedance *Z* and the longitudinal acoustic velocity *v*_*a*_ are adjusted to fit the *f*_*p*_ of the impedance curve. The intrinsic electromechanical coupling factor $K_t^2$ is then adjusted to fit the *f*_*s*_ of the curve. The fitted impedance of the Mason model and the measured impedance curve are shown in Fig. 5(b). With the equivalent Mason circuit, we can achieve a very consistent fitting result with the measured impedance. Furthermore, the unknown parameters (*ρ*, *e*_33_, $C_{33}^E$) can be obtained by^26,27^.

7 $$\rho = \frac{Z}{{v_a}}$$

8 $$c_{33}^D = \rho v_a^2$$

9 $$e_{33} = \sqrt {K_t^2c_{33}^D\varepsilon _r\varepsilon _0}$$

10 $$C_{33}^E = c_{33}^D - \frac{{e_{33}^2}}{{\varepsilon _r\varepsilon _0}}$$

where *ρ* is the mass density, $c_{33}^D$ is the stiffened elastic constant, *e*_33_ is the piezoelectric stress constant, $C_{33}^E$ is the elastic constant, and $\varepsilon _{{{\mathrm{r}}}}$ is the relative dielectric permittivity. The $\varepsilon _{{{\mathrm{r}}}}$ of the Al_0.8_Sc_0.2_N film is separately extracted from the static FBAR capacitance *C*_*0*_, applying the Formula *C*_*0*_ = $\varepsilon _{{{\mathrm{r}}}}\varepsilon _0A/d$. Here, *A* is the active FBAR area, and *d* is the Al_0.8_Sc_0.2_N film thickness. Table 2 lists the extracted film parameters of Al_0.8_Sc_0.2_N, highlighting a larger *e*_33_ of 2.08 C/m^2^ than that of AlN (1.55 C/m^2^)^28^.

**Table 2:** Extracted film parameters

Material	${{\boldsymbol{K}}_{\boldsymbol{t}}^{\boldsymbol{2}}}$	${{\boldsymbol{\varepsilon}} _{{{\boldsymbol{r}}}}}$	*ρ* (kg/m^3^)	*v*_*a*_ (m/s)	${\boldsymbol{C}}_{\boldsymbol{33}}^{\boldsymbol{E}}$ (Gpa)	${\boldsymbol{e}}_{\boldsymbol{33}}$ (C/m^2^)
Al_0.8_Sc_0.2_N	12.4	13.42	3560	9089	258	2.08

**Fig. 6:**
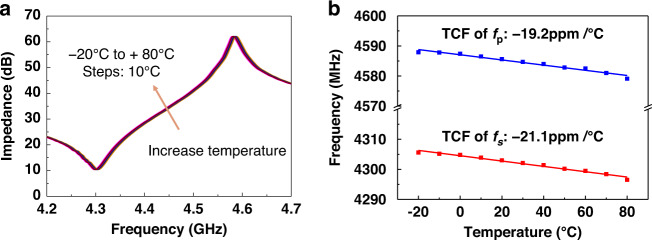
Temperature characteristics of the fabricated Al_0.8_Sc_0.2_N-based FBAR. **a** Frequency responses of the fabricated Al_0.8_Sc_0.2_N-based FBAR at temperatures from −20 °C to 80 °C. **b** Measured TCF of the proposed FBAR.

By measuring the frequency responses at different temperatures, the temperature coefficients *f*_*s*_ and *f*_*p*_ of the proposed Al_0.8_Sc_0.2_N-based FBAR are found to be −19.2 ppm/°C and −21.1 ppm/°C, respectively, as shown in Fig. 6. TCF is usually an intrinsic property of materials that is dependent on the growth conditions. The TCF of the proposed device is comparable with previously reported values^29,30^. The difference in TCF between *f*_*s*_ and *f*_*p*_ is caused by a slight shift in $K_t^2$ since the change in temperature may induce thermal stress in the thin film.

**Fig. 7:**
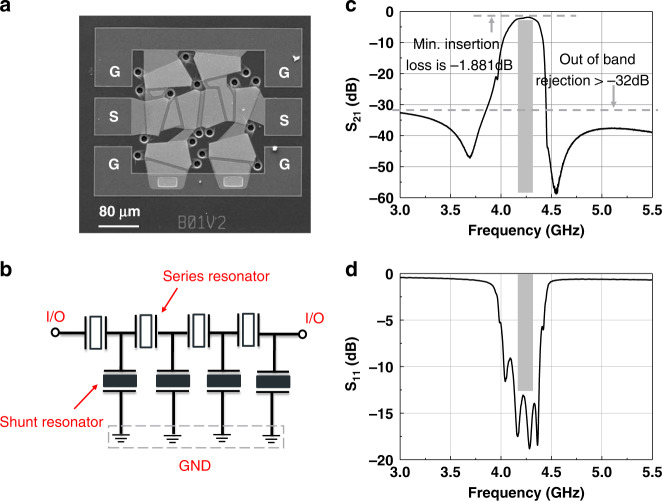
Experimental results of the fabricated FBAR filter. **a** SEM image for the fabricated FBAR filter. **b** Schematic circuit design of the FBAR filter. **c** Measured transmission response of the fabricated FBAR filter. **d** Measured return loss of the fabricated FBAR filter.

Figure 7(a) and (b) show the SEM image and schematic circuit of the fabricated filter. It consists of 8 elements, including 4 series and 4 shunt resonators. To achieve passband transmit characteristics, a 37 nm Mo mass loading layer is added to the shunt resonator to make its resonant frequency lower than the series resonator. Table 3 summarizes the relevant measured and extracted parameters of the series and shunt resonators used in the filter. Moreover, additional Mo layers are constructed on the interconnect line between each adjacent resonator to improve the performance of the filter. A plot of the measured filter response is shown in Fig. 7(c) and (d). The filter shows a center frequency of 4.24 GHz, a −3 dB bandwidth of 215 MHz, a small IL of 1.881 dB, and a rejection >32 dB. The measured return loss at the center frequency is below −12 dB, indicating a good match to 50 Ω without any helper circuits.

**Table 3:** Measured and extracted FBAR parameters.

Resonator	*A* (μm^2^)	*C*_*0*_ (pF)	*f*_*s*_ (GHz)	*Q*_*s*_	*f*_*p*_ (GHz)	*Q*_*p*_	*K*_*eff*_^2^ (%)
Series	3281	0.75	4.301	150	4.588	318	14.5
Shunt	3794	0.88	4.001	130	4.274	202	14.8

## Conclusion

In this paper, an Al_0.8_Sc_0.2_N-based FBAR is investigated for the design of acoustic MEMS filters. The proposed FBAR devices are yielded by a seven-mask layer wafer process, including sputtering deposition, ICP etching, and cavity release. Characterization of the microstructural and crystal structure properties of the sputtered Al_0.8_Sc_0.2_N thin film is performed. The results show that the Al_0.8_Sc_0.2_N layer has a good c-axis preferential crystal orientation. For the FBAR devices based on the Al_0.8_Sc_0.2_N film with a large *e*_33_ (2.08 C/m^2^), a large *K*_*eff*_^2^ of 14.5% and an improved FOM of 62 are simultaneously obtained. Furthermore, the temperature characteristics of the Al_0.8_Sc_0.2_N-based FBAR are studied. The TCFs of *f*_*s*_ and *f*_*p*_ of the proposed resonator are found to be −19.2 ppm/°C and −21.1 ppm/°C, respectively, indicating excellent temperature stability. The fabricated filter based on the above resonators has a center frequency of 4.24 GHz, a −3 dB bandwidth of 215 MHz, a small IL of 1.881 dB, and a rejection >32 dB, showing the strong potential for 5G RF front-end applications.
